# Bielectrode Strategy for Determination of CYP2E1 Catalytic Activity: Electrodes with Bactosomes and Voltammetric Determination of 6-Hydroxychlorzoxazone

**DOI:** 10.3390/biomedicines12010152

**Published:** 2024-01-08

**Authors:** Alexey V. Kuzikov, Rami A. Masamrekh, Tatiana A. Filippova, Anastasiya M. Tumilovich, Natallia V. Strushkevich, Andrei A. Gilep, Yulia Yu. Khudoklinova, Victoria V. Shumyantseva

**Affiliations:** 1 Institute of Biomedical Chemistry, 10, Pogodinskaya Street, 119121 Moscow, Russia; rami.masamreh@yandex.ru (R.A.M.); lipivif@gmail.com (T.A.F.); agilep@yahoo.com (A.A.G.); viktoria.shumyantseva@ibmc.msk.ru (V.V.S.); 2 Department of Biochemistry, Faculty of Biomedicine, Pirogov Russian National Research Medical University, 1, Ostrovityanova Street, 117997 Moscow, Russia; hudoklinova@mail.ru; 3 Institute of Bioorganic Chemistry NASB, 5 Building 2, V.F. Kuprevich Street, 220084 Minsk, Belarus; tumilovicham@iboch.by (A.M.T.); natstrush@gmail.com (N.V.S.)

**Keywords:** Bactosomes, chlorzoxazone, CYP2E1, screen-printed electrodes, voltammetry, 6-hydroxychlorzoxazone

## Abstract

We describe a bielectrode system for evaluation of the electrocatalytic activity of cytochrome P450 2E1 (CYP2E1) towards chlorzoxazone. One electrode of the system was employed to immobilize Bactosomes with human CYP2E1, cytochrome P450 reductase (CPR), and cytochrome *b*_5_ (cyt *b*_5_). The second electrode was used to quantify CYP2E1-produced 6-hydroxychlorzoxazone by its direct electrochemical oxidation, registered using square-wave voltammetry. Using this system, we determined the steady-state kinetic parameters of chlorzoxazone hydroxylation by CYP2E1 of Bactosomes immobilized on the electrode: the maximal reaction rate (*V*_max_) was 1.64 ± 0.08 min^−1^, and the Michaelis constant (*K*_M_) was 78 ± 9 μM. We studied the electrochemical characteristics of immobilized Bactosomes and have revealed that electron transfer from the electrode occurs both to the flavin prosthetic groups of CPR and the heme iron ions of CYP2E1 and cyt *b*_5_. Additionally, it has been demonstrated that CPR has the capacity to activate CYP2E1 electrocatalytic activity towards chlorzoxazone, likely through intermolecular electron transfer from the electrochemically reduced form of CPR to the CYP2E1 heme iron ion.

## 1. Introduction

The cytochrome P450 (CYP) enzyme superfamily is one of the major enzyme systems catalyzing the biotransformation of xenobiotics, including pharmaceuticals and toxic compounds in the environment, and the metabolism of endogenous compounds [1,2]. Conventional methods employed to assess the activity of cytochromes P450, relying on chromatography and mass spectrometry, despite their wide use, involve multiple steps, are labor-intensive, and necessitate additional steps to eliminate from samples proteins and other molecules complicating the analysis. Moreover, they demand substantial quantities of the enzyme to attain the limit of detection for the resultant metabolites [3,4].

For studying the functional characteristics of hemoproteins, electrochemical methods are widely used, making it possible to investigate the reduction potentials, thermodynamics, and kinetics of chemical reactions related to electron transfer [5,6,7,8,9]. The utilization of electrochemical systems is one of the contemporary approaches for evaluating the activity of cytochromes P450. In such systems, an electrode functions as an electron donor to reduce the heme iron ion of cytochrome P450, thus initiating a catalytic reaction towards the substrate. This approach eliminates the requirement for natural electron donors like NADPH [10,11,12]. The immobilization of cytochromes P450 on an electrode can enhance their stability and lower the enzyme quantity needed in studies [13]. An array of electrochemical systems based on recombinant cytochromes P450 has been developed to date [14,15,16,17]. Previously, we proposed a technique for optimizing electrochemical systems designed to assess the activity of cytochromes P450, involving a bielectrode strategy. This strategy entails the use of two electrodes, with one serving to immobilize the recombinant enzyme and the other to detect the metabolites generated during the reaction by their direct electrochemical oxidation [18,19,20,21,22]. Such systems allowed us to sidestep the requirement for multi-step sample preparation procedures and the need to separate metabolites and substrates.

Along with the creation of electrochemical systems based on recombinant cytochromes P450, there has been significant progress in the development of electrochemical systems based on human microsomes immobilized on electrodes or microsomes obtained from transgenic organisms. These include microsomes derived from insect cells infected with recombinant baculovirus containing a human CYP isoenzyme, as well as human cytochrome P450 reductase (Baculosomes™, Thermo Fisher Scientific, Carlsbad, CA, USA; Supersomes™, Corning Incorporated, Tewksbury, MA, USA) and membrane fractions derived from *E. coli* producing individual cytochrome P450 isoenzymes and components of the human monooxygenase system (Bactosomes^®^, Cypex Ltd., Edinburgh, UK). A notable advantage of such systems is that they eliminate the need to isolate cytochrome P450 from the membrane environment, thereby preventing enzyme inactivation. To immobilize microsomes or membrane fractions containing monooxygenase system components on electrodes, technologies similar in principle to those used for immobilizing recombinant cytochromes P450 are applied. Unlike recombinant enzyme forms, which can lose their stability and activity when adsorbed on unmodified electrodes, membrane-bound cytochromes P450 can be effectively adsorbed onto electrodes while preserving their catalytic activity [23,24]. Microsomes can be immobilized on electrodes modified with thin hydrophobic films of organic compounds, such as different thiolates [25,26]. Utilizing the negative charge of phospholipids in biological membranes enables immobilization through electrostatic interaction with electrodes modified with compounds bearing a positive charge, such as cysteamine [27] or magnetic nanoparticles (Fe_3_O_4_) functionalized with positively charged amines [28]. Electrochemical systems have been developed that utilize layer-by-layer technology for sequential deposition of films using substances like polyethylenimine, polystyrene sulfonate, and microsomes [29]. Immobilization of microsomes is also carried out on electrodes modified with nanocomposite materials based on colloidal gold and graphene stabilized with poly(diallyldimethylammonium chloride) [30]. Encapsulation of enzymes in polymers and gels to immobilize them on electrodes has also been used for microsomes [31,32]. Microsomes can be immobilized on electrodes modified with carbon nanotubes [33,34].

**Scheme 1:**
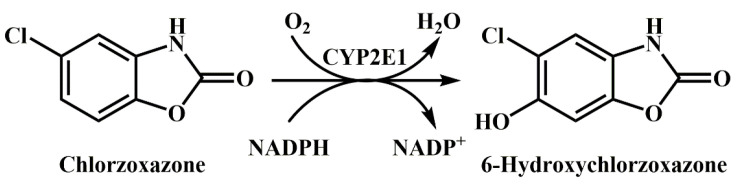
Reaction converting chlorzoxazone into 6-hydroxychlorzoxazone, catalyzed by CYP2E1.

Cytochrome P450 family 2, subfamily E, member 1 (CYP2E1) and alcohol dehydrogenase are involved in the oxidation of ethanol [35,36,37,38]. Additionally, CYP2E1 is responsible for metabolizing less than 5% of commonly prescribed drugs, some of which, when metabolized by this enzyme, yield potentially toxic compounds [39,40]. Recognizing CYP2E1 as a target for decreasing the toxicity resulting from alcohol metabolism and drug–alcohol interactions has sparked interest in the search for specific inhibitors of this enzyme [41]. One of the substrates of CYP2E1 is chlorzoxazone, a centrally acting muscle relaxant. When metabolized by this enzyme, chlorzoxazone undergoes a transformation, resulting in the production of 6-hydroxychlorzoxazone [42] (Scheme 1). Chlorzoxazone serves as a marker substrate for CYP2E1 and is commonly employed to assess the activity of this enzyme [43].

Conventional methods for assessing CYP2E1’s 6-hydroxylase activity towards chlorzoxazone in microsomal or reconstituted systems rely on high-performance liquid chromatography with UV detection [42,44] or mass spectrometry analysis of the resulting metabolite.

In this study, we aimed to design an electrochemical system for evaluating the activity of membrane-bound cytochromes P450, using CYP2E1-containing Bactosomes as an example. To achieve this, we employed a bielectrode strategy. In this approach, one electrode was utilized to immobilize Bactosomes containing human CYP2E1, cytochrome P450 reductase (CPR), and human cytochrome *b*_5_ (cyt *b*_5_), while the other electrode served to quantify the metabolite of the CYP2E1 marker substrate chlorzoxazone, namely, 6-hydroxychlorzoxazone, by its electrochemical oxidation. In this system, the electrode used to immobilize Bactosomes acted as the source of electrons to reduce CYP2E1 and initiate the catalytic reaction. Importantly, CYP2E1 was located in the membrane environment and surrounded by its natural redox partner proteins (CPR and cyt *b*_5_), ensuring maximal enzyme stability. The inclusion of cyt *b*_5_ in the system allowed for the creation of an enzyme system with maximal activity, as cyt *b*_5_ is known to enhance CYP2E1 catalytic function [45,46,47]. Quantitative voltammetric determination of the resulting metabolite (6-hydroxychlorzoxazone) by its direct electrochemical oxidation at potentials distinct from the substrate (chlorzoxazone) oxidation potential eliminated the need for additional metabolite isolation steps.

In summary, this study aimed to develop a bielectrode system for assessing the catalytic activity of cytochromes P450, utilizing electrodes with immobilized Bactosomes and voltammetric analysis of the metabolite as demonstrated through the CYP2E1 hydroxylase activity towards chlorzoxazone.

## 2. Materials and Methods

### 2.1. Reagents

The following reagents were purchased from Sigma-Aldrich (Waltham, MA, USA): catalase from bovine liver (2000–5000 units/mg protein), didodecyldimethylammonium bromide (DDAB, 98%), potassium phosphate monobasic (≥99%), potassium phosphate dibasic trihydrate (≥99%), chlorzoxazone (≥98%), and 6-hydroxychlorzoxazone (≥98%). Potassium hydroxide (≥99%) was purchased from Component-reaktiv (Moscow, Russia), and sodium chloride (99.9%) was obtained from Acros Organics (Carlsbad, CA, USA). Chloroform (chemically pure, stabilized with 0.6–1 wt.% ethanol), methylene chloride (chemically pure), and petroleum ether (boiling range 50–70 °C) were provided by ECOS-1 (Moscow, Russia). Ethyl acetate (HPLC grade) was purchased from Scharlab (Barcelona, Spain). Human CYP2E1BR Bactosomes (Cat # CYP036) with a protein concentration equal to 17.5 mg/mL (4.3 µM cytochrome P450 and CYP-reductase, co-expressed in *E. coli* with cytochrome *c* reductase activity of 673 nmol/min/mg protein, supplemented with purified human cytochrome *b*_5_ at a 21.4 µM concentration) were purchased from Cypex Ltd. (Edinburgh, UK). The maximal rate of the 6-hydroxylation of chlorzoxazone (*V*_max_) is 9.3 min^−1^ and the Michaelis constant (*K*_M_) is 75 µM, according to the product information sheet. Recombinant human CYP2E1 with a protein concentration of 72 μM in 50 mM potassium phosphate buffer, pH 7.4, containing 400 mM NaCl, 1 mM EDTA, and 20% glycerol, was obtained in accordance with the previously described procedure [48]. Microsomal human cytochrome *b*_5_ (cyt *b*_5_) with a protein concentration of 158 μM in 400 mM potassium phosphate buffer, pH 7.4, containing 0.2% sodium cholate and 20% glycerol, was obtained in accordance with the previously described procedure [49]. Recombinant rat CPR with a protein concentration of 165.9 μM in 50 mM Tris buffer, pH 7.5, containing 0.5 mM EDTA, 0.1 mM DTT, and 20% glycerol, was obtained in accordance with the previously described procedure [50].

Stock solutions of chlorzoxazone and 6-hydroxychlorzoxazone in 60 mM KOH were used in this research.

### 2.2. Electrodes

To investigate the electrochemical characteristics of chlorzoxazone and 6-hydroxychlorzoxazone, we employed screen-printed electrodes (SPEs) with graphite working (d = 2 mm) and auxiliary electrodes, and a silver–silver chloride reference electrode (Ag/AgCl) sourced from ColorElectronics (Moscow, Russia).

For the exploration of the electrochemical properties of Bactosomes, CYP2E1, cyt *b*_5_, and CPR, we utilized SPEs featuring a graphite working electrode with a larger diameter (d = 4 mm), graphite auxiliary electrode, and an Ag/AgCl reference electrode obtained from Qingdao Poten Technology Co., Ltd. (Qingdao, China). To immobilize Bactosomes, CYP2E1, cyt *b*_5_, or CPR, the working electrodes were modified with 3 μL of 0.1 M didodecyldimethylammonium bromide (DDAB) in chloroform. Following the evaporation of chloroform, 1.5 μL Bactosomes, 0.5 μL 72 μM CYP2E1, 1.5 μL 158 μM cyt *b*_5_, 1.5 μL 165.9 μM CPR, or 1.5 μL of a mixture comprised of 0.46 μL 72 μM CYP2E1 and 1.04 μL 158 μM cyt *b*_5_ (the molar ratio in the mixture was 1:5) was applied onto the modified surface of the working electrodes. The resulting electrodes were held for 12 h at 4 °C within a humid chamber to prevent desiccation of the immobilized biological material.

All potentials in this study are referred to the Ag/AgCl reference electrode.

Electrochemical experiments were conducted at 24 ± 2 °C unless otherwise specified.

### 2.3. Electrochemical Analysis of Bactosomes, CYP2E1, cyt b_5_, and CPR

Cyclic voltammetry (CV) was employed to explore the electrochemical characteristics of Bactosomes, CYP2E1, cyt *b*_5_, and CPR immobilized on the electrodes. For investigations conducted under anaerobic conditions, electrodes with immobilized Bactosomes, CYP2E1, cyt *b*_5_ (or the mixture of CYP2E1 and cyt *b*_5_), or CPR were placed in a plastic cell filled with 1 mL of 100 mM potassium phosphate buffer, pH 7.4, containing 50 mM NaCl. The cell was subsequently sealed hermetically, and argon was passed through for 30 min. Cyclic voltammograms were then registered within the potential range from 0.1 to −0.75 V, with scan rates ranging from 10 to 200 mV/s.

The amount of electroactive hemoproteins was calculated according to Equation (1):

(1) $$The\;amount\;of\;electroactive\;hemoproteins=\frac{Q}{nF},$$

where *Q* is the electric charge (C) calculated from integration of peaks of cyclic voltammograms, *n* is the number of electrons involved in the electrochemical process (for heme iron ions it is equal to 1), and *F* is the Faraday constant (96,485 C/mol).

Differential pulse voltammetry (DPV) was utilized to investigate Bactosomes and CPR immobilized on DDAB-modified SPEs. Differential pulse voltammograms were registered under anaerobic conditions in the potential range of 0.1 to −0.75 V, at the modulation amplitude 20 mV, step potential 5 mV, interval time 500 ms, and modulation time 50 ms.

### 2.4. Electrochemical Analysis of Chlorzoxazone and 6-Hydroxychlorzoxazone

The electrochemical properties of chlorzoxazone and 6-hydroxychlorzoxazone were studied by cyclic voltammetry. To do this, 60 μL of 100 mM potassium phosphate buffer, pH 7.4, containing 50 mM NaCl and 50 μM chlorzoxazone or 50 μM 6-hydroxychlorzoxazone or an equimolar mixture of chlorzoxazone and 6-hydroxychlorzoxazone (50 μM of each compound) was applied to the surface of SPEs (ColorElectronics). Cyclic voltammograms were then registered within the potential range of 0 to 1.2 V, with a scan rate of 100 mV/s.

Square-wave voltammetry (SWV) was used to quantify 6-hydroxychlorzoxazone. To obtain a calibration curve for the oxidation current of 6-hydroxychlorzoxazone on its concentration, we applied 60 μL of 100 mM potassium phosphate buffer, pH 7.4, containing 50 mM NaCl, and different concentrations of 6-hydroxychlorzoxazone (0.1–1 μM) to the SPE surface, followed by registration of square-wave voltammograms in the potential range of 0 to 1.2 V, at a frequency of 25 Hz, amplitude of 40 mV and potential step of 5 mV.

### 2.5. Determination of the Electrocatalytic Activity of Bactosomes on Modified Electrodes by Electrochemical Analysis of 6-Hydroxychlorzoxazone

To determine the electrocatalytic activity of Bactosomes immobilized on SPEs modified with DDAB towards chlorzoxazone, the SPEs were placed in a cell filled with 1 mL of 100 mM potassium phosphate buffer, pH 7.4, containing 50 mM NaCl for 5 min at 37 °C to wash off Bactosomes not bound to the modified surface of the electrode. SPEs with immobilized Bactosomes were then placed in a cell filled with 900 μL of 100 mM potassium phosphate buffer, pH 7.4, containing 50 mM NaCl and various concentrations of chlorzoxazone (10–500 μM). In experiments designed to assess the impact of the working electrode potential on the initial rate of 6-hydroxychlorzoxazone formation during the electrocatalytic reaction, the aforementioned buffer solution also contained 280 units/mL of bovine liver catalase. The electrocatalytic reactions were conducted at a fixed potential of either −0.35 V or −0.55 V for 30 to 90 min, with the incubation mixture continuously stirred using a magnetic stirrer at 37 °C. To determine the concentration of the yielded 6-hydroxychlorzoxazone after the electrocatalytic reaction, 60 μL of the incubation mixture was applied to the unmodified SPE (ColorElectronics). Subsequently, a square-wave voltammogram was registered using the parameters specified above. The concentration of the generated 6-hydroxychlorzoxazone was determined using the calibration curve equation, based on the oxidation peak current registered in the 0.2 V potential range as a function of 6-hydroxychlorzoxazone concentration. The initial rate of 6-hydroxychlorzoxazone formation (*V*) at various chlorzoxazone concentrations was determined from a plot of the quantity of 6-hydroxychlorzoxazone produced versus the electrocatalytic reaction time. The maximal rate of 6-hydroxychlorzoxazone formation (*V*_max_) and the Michaelis constant (*K*_M_) were calculated from the hyperbolic dependence of *V* on chlorzoxazone concentration, according to the Michaelis–Menten equation, Equation (2):

(2) $$V=\frac{V_{\max}\left[S\right]}{K_{M}+\left[S\right]},$$

where *V* is the formation rate of 6-hydroxychlorzoxazone, min^−1^, [S] is the concentration of the substrate (chlorzoxazone), M, *K*_M_ is the Michaelis constant, M, *V*_max_ is the maximal formation rate of 6-hydroxychlorzoxazone, min^−1^.

### 2.6. Determination of the Electrocatalytic Activity of Bactosomes on Modified Electrodes by Thin-Layer Chromatography and Absorbance Spectroscopy of 6-Hydroxychlorzoxazone

After the electrocatalytic reaction for 90 min, 450 μL of the incubation mixture was pooled into a test tube and mixed with 900 μL of methylene chloride followed by centrifugation for 5 min at 12,100× *g*. After the centrifugation, the lower (organic) phase was pooled into another test tube and the remaining higher phase was once again mixed with 900 μL of methylene chloride followed by centrifugation under the abovementioned conditions. After the second centrifugation, the lower phase was combined with the organic phase obtained after the first centrifugation and the whole volume was vaporized at 38 °C under the flow of argon. The vaporized samples were resolubilized with 20 μL of ethanol and spotted onto aluminum thin-layer chromatography plates coated with silica gel and fluorescent indicator F254 (TLC Silica gel 60, F254, Merck, Darmstadt, Germany). As a control, 1 μL of 2.5–10 mM chlorzoxazone and 1 μL of 2.5–10 mM 6-hydroxychlorzoxazone in ethanol were also applied onto the plates. Chromatography was performed with petroleum ether and ethyl acetate (1:1, *v*/*v*) mixture as the moving phase in a glass chamber. After chromatography, the plates were dried at room temperature and, for better visualization of 6-hydroxychlorzoxazone spots, the plates were placed into a desiccator saturated with iodine vapors for 2 min exactly. Visualization of the separated compounds on the TLC plates was performed using an ultraviolet lamp (Vilber Lourmat, Marne-la-Vallée, France) at the wavelength of 254 nm. The spots on the plates identified as 6-hydroxychlorzoxazone in the experimental and control tracks were isolated and eluted with 600 μL each. The silica gel flakes were sedimented by centrifugation for 5 min at 12,100× *g*. The absorbance spectra of 6-hydroxychlorzoxazone in the supernatant were registered in a quartz cuvette at the wavelength range of 230–350 nm using a Shimadzu UV-1900 spectrophotometer (Shimadzu Corporation, Kyoto, Japan) equipped with UV Probe 2.70 software. The concentration of 6-hydroxychlorzoxazone was determined using the calibration dependence of the absorbance maximum of 6-hydroxychlorzoxazone at the wavelength range of 280–300 nm on the compound’s concentration in the standard solutions.

## 3. Results and Discussion

### 3.1. Investigation of the Electrochemical Properties of Bactosomes Immobilized on Electrodes

To immobilize Bactosomes on an electrode, as a surface modifier we employed DDAB, a commonly used compound in cytochromes P450 bioelectrochemistry. DDAB forms a stable bilayer at room temperature and has a positively charged nitrogen atom that interacts electrostatically with the negatively charged surface of the graphite electrode [11,51]. We hypothesize that Bactosomes can interact with the modified electrode through hydrophobic interactions between the components of Bactosomes and the aliphatic chains of DDAB, as well as through electrostatic interactions between the negatively charged phosphate groups of membrane phospholipids of Bactosomes and the positively charged nitrogen atom of DDAB.

**Figure 1:**
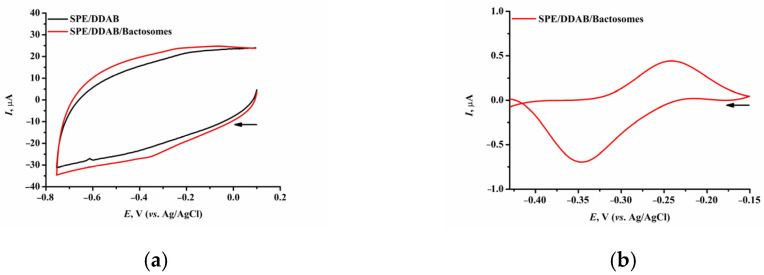
Cyclic voltammograms of SPEs modified with DDAB (▬) and immobilized Bactosomes (▬) registered in 100 mM potassium phosphate buffer, pH 7.4, containing 50 mM NaCl saturated with argon (anaerobic conditions) (**a**) and cyclic voltammogram obtained after subtracting the cyclic voltammogram of DDAB-modified SPE from the cyclic voltammogram of DDAB-modified SPE with immobilized Bactosomes (**b**). The voltammograms were registered at a 50 mV/s scan rate.

The cyclic voltammogram of the electrode with immobilized Bactosomes in 100 mM potassium phosphate buffer, pH 7.4, containing 50 mM NaCl under anaerobic conditions (argon-saturated), exhibited a reduction peak (*E*_c_) at −0.345 ± 0.006 V and an oxidation peak (*E*_a_) at −0.251 ± 0.013 V (Figure 1). The midpoint potential value (*E*_m_), calculated as the average of the reduction and oxidation peak potentials, was determined to be −0.298 ± 0.010 V. Given that the studied Bactosomes contain the hemoproteins CYP2E1 and cyt *b*_5_, it is reasonable to assume that the observed redox pair of peaks corresponds to the reduction and oxidation processes of the heme iron ions in both CYP2E1 and cyt *b*_5_. The amounts of electroactive CYP2E1 and cyt *b*_5_ were calculated using Equation (1), accounting for the molar ratios of those hemoproteins in Bactosomes, as 9 pmol and 45 pmol, respectively. Since the redox process for the flavin component of CPR, which is part of the Bactosomes, could not be detected, likely due to the limited sensitivity of cyclic voltammetry, we employed the differential pulse voltammetry method to identify the reduction and oxidation peaks of CPR flavins.

**Figure 2:**
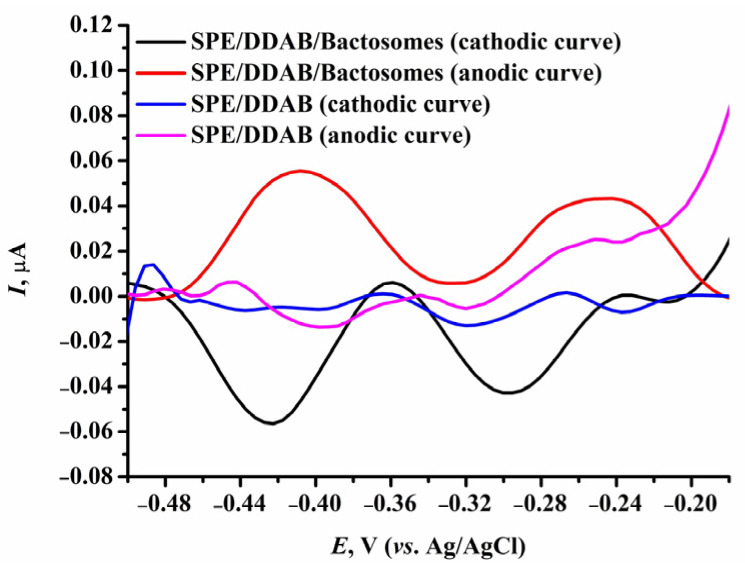
Differential pulse voltammograms of SPEs modified with DDAB (cathodic curve (▬) and anodic curve (▬)) and with immobilized Bactosomes (cathodic curve (▬) and anodic curve (▬)) registered in 100 mM potassium phosphate buffer, pH 7.4, containing 50 mM NaCl saturated with argon (anaerobic conditions). Modulation amplitude 20 mV, step potential 5 mV, interval time 500 ms, modulation time 50 ms.

As can be seen in Figure 2, the differential pulse voltammograms are characterized by a reduction peak at *E*_c_ −0.307 ± 0.012 V and an oxidation peak at *E*_a_ −0.261 ± 0.020 V, and the calculated value of *E*_m_, −0.284 ± 0.011 V, is close to the similar parameter obtained using cyclic voltammetry. Thus, this pair of peaks characterizes the redox process for the iron ions of the heme groups of CYP2E1 and cyt *b*_5_. Also, the differential pulse voltammogram exhibits a pair of peaks at *E*_c_ −0.454 ± 0.038 V and *E*_a_ −0.410 ± 0.023 V with *E*_m_ −0.432 ± 0.026 V. Previously, in the works of Nerimetla et al. for Bactosomes containing only CPR electrostatically adsorbed on a cysteamine monolayer of gold-coated quartz crystals or electrostatically immobilized on edge-plane pyrolytic graphite electrodes with amine-functionalized magnetic nanoparticles, the formal potential at pH 7.0 was determined to be −0.450 ± 0.038 V (vs. Ag/AgCl) [27] or −0.47 ± 0.02 V (vs. Ag/AgCl) [28], respectively. Thus, the *E*_m_ obtained for the second pair of Bactosomes peaks is in good agreement with a similar parameter previously obtained by other authors for CPR.

We also investigated the electrochemical characteristics of recombinant CYP2E1 (Figure S1 in the Supplementary Materials) and cyt *b*_5_ (Figure S2 in the Supplementary Materials), as well as a combination of both (in a 1:5 molar ratio) (Figure S3 in the Supplementary Materials), immobilized on DDAB-modified electrodes. For CYP2E1, the *E*_c_ and *E*_a_ values were registered at −0.301 ± 0.007 V and −0.263 ± 0.008 V, respectively, and the *E*_m_ value was calculated as −0.282 ± 0.005 V. Consequently, the *E*_m_ value for CYP2E1 exceeded that of Bactosomes by 16 mV. In the case of cyt *b*_5_, the *E*_c_ and *E*_a_ values were −0.288 ± 0.009 V and −0.222 ± 0.017 V, respectively, with *E*_m_ of −0.255 ± 0.005 V. Thus, the *E*_m_ for cyt *b*_5_ exceeded the *E*_m_ for Bactosomes by 43 mV and for CYP2E1 by 27 mV. When analyzing a mixture of recombinant CYP2E1 and cyt *b*_5_, we also observed a redox couple at potentials of −0.310 ± 0.014 V and −0.242 ± 0.007 V, with *E*_m_ of −0.276 ± 0.011 V. This *E*_m_ value for the mixture was between the *E*_m_ values for recombinant CYP2E1 and cyt *b*_5_. Additionally, we conducted an examination of recombinant rat CPR immobilized on an electrode using differential pulse voltammetry (Figure S4 in the Supplementary Materials). As depicted in Figure S4 in the Supplementary Materials, the cathodic curve displayed a reduction peak at *E*_c_ −0.421 ± 0.003 V, while the anodic curve exhibited an oxidation peak at *E*_a_ −0.402 ± 0.012 V. The *E*_m_ value was determined as −0.412 ± 0.005 V, closely resembling similar parameters obtained using differential pulse voltammetry for the second pair of peaks associated with the redox process of the flavin components in the CPR of Bactosomes.

Analysis of the data obtained suggests that the more negative *E*_m_ value for the pair of peaks obtained by cyclic voltammetry for Bactosomes, compared with the same parameter for recombinant CYP2E1, cyt *b*_5_, and their mixture, may be a consequence of the effect on electron transfer of CPR in Bactosomes. It is likely that CPR, CYP2E1, and cyt *b*_5_ are the acceptors of electrons from the electrode.

The electrochemical characteristics of Bactosomes, CYP2E1, cyt *b*_5_, the CYP2E1 + cyt *b*_5_ mixture, and CPR registered under anaerobic conditions are summarized in Table 1.

It is noteworthy that in pioneering investigations of the electrochemical properties of membrane-bound components of the monooxygenase system, the researchers reported the redox activity of CPR only. For instance, Sultana et al. documented a pair of peaks with an *E*_m_ of −0.49 V (vs. SCE) for CYP1A2- and CYP3A4-containing microsomes, which were adsorbed using a layer-by-layer method onto electrodes modified by the successive deposition of polymer films based on polyethylenimine and polystyrene sulfonate. These authors characterized this redox process as being associated with CPR [29]. At the same time, *E*_m_ values of −0.31 V and −0.32 V, respectively, were determined for recombinant CYP1A2 and CYP3A4. Consequently, these findings suggest that CPR serves as the primary acceptor of electrons from the electrode in microsomes. Additionally, these authors observed the conversion of styrene to styrene oxide during an electrocatalytic reaction at the potential at which CPR reduction occurs. Nerimetla et al. also noted a pair of peaks with a formal potential of −450 ± 40 mV (vs. Ag/AgCl) for Bactosomes that contained CYP2C9 and CPR, which were electrostatically adsorbed onto a cysteamine self-assembling monolayer of Au-infused quartz crystal. In comparison, the formal potential values for recombinant CYP2C9 and CPR were determined to be −310 ± 20 mV and −450 ± 38 mV, respectively [27]. Furthermore, during the adsorption of human microsomes onto various carbon electrodes, Walgama et al. observed a pair of peaks with formal potential values ranging from −0.450 to −0.470 V (vs. Ag/AgCl), consistent with the formal potential characteristic of CPR [23]. Krishnan et al. also confirmed electron transfer from the electrochemically reduced form of CPR to CYP1A2 or CYP2E1 sequentially deposited on polyionic gels [52].

On the other hand, in a study conducted by Xu et al., where they immobilized CYP2C9 isoenzyme microsomes with cytochrome P450 reductase on a glassy carbon electrode modified with a nanocomposite material comprising indium tin oxide nanoparticles and chitosan at pH 7.4, the researchers observed a pair of peaks at *E*_c_ −0.404 V and *E*_a_ −0.382 V (vs. SCE). The authors concluded that this pair of peaks reflects a redox process involving the heme in CYP2C9 and the flavin prosthetic groups in CPR [31]. When examining the electrochemical properties of flavocytochrome P450 BM3 containing both reductase and heme domains, immobilized on an electrode using DDAB, under anaerobic conditions using cyclic voltammetry, multiple studies documented two pairs of peaks, representing redox processes associated with both the flavin and heme groups [53,54].

We assume that this difference in the observed mechanisms of electron transfer in systems concurrently involving CPR and cytochromes P450 can be attributed to the use of different types and modifications of electrodes for enzyme immobilization. Consequently, this affects the availability of active sites for the direct electrochemical reduction of their cofactors.

**Figure 3:**
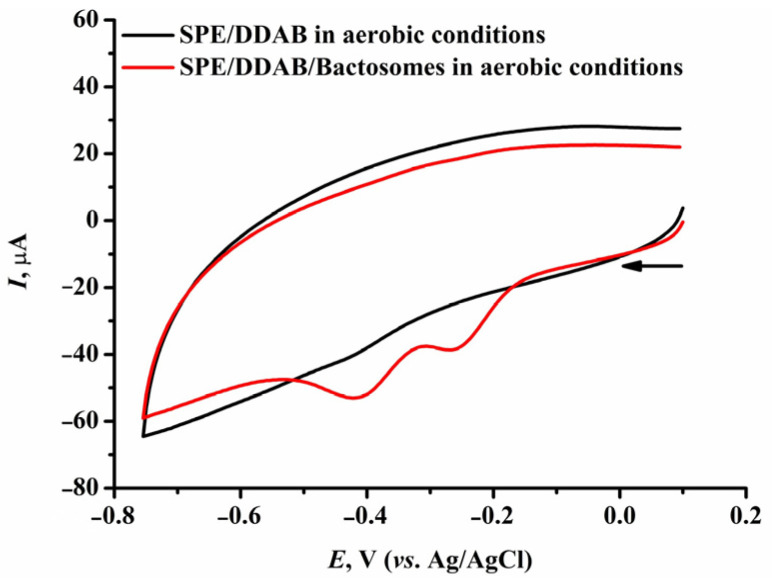
Cyclic voltammograms of SPEs modified with DDAB (▬) and immobilized Bactosomes (▬) registered in 100 mM potassium phosphate buffer, pH 7.4, containing 50 mM NaCl (aerobic conditions). The voltammograms were registered at a 50 mV/s scan rate.

Moreover, we conducted further analysis of the electrochemical characteristics of Bactosomes, recombinant CYP2E1, cyt *b*_5_, their mixture (CYP2E1 + cyt *b*_5_), and CPR under aerobic conditions. The cyclic voltammogram of the electrode with immobilized Bactosomes exhibited two reduction peaks at −0.262 ± 0.007 V and −0.418 ± 0.017 V (Figure 3). Previously, Carrara et al. observed a split in the reduction peak when investigating the electrochemical properties of CYP1A2-containing microsomes. After mathematical processing, the authors were able to separate two reduction peaks at −0.380 V and −0.560 V (vs. Ag/AgCl), which, according to their interpretation, correspond to the reduction processes of CYP1A2 and CPR [33].

We assume that the reduction peaks registered under aerobic conditions correspond to reduction of the heme iron ions of the hemoproteins CYP2E1 and cyt *b*_5_ of the Bactosomes (−0.262 ± 0.007 V), and the flavin prosthetic groups of CPR (−0.418 ± 0.017 V) of the Bactosomes. To prove this assumption, we analyzed the electrochemical properties of recombinant CYP2E1, cyt *b*_5_, and CPR under aerobic conditions. In the case of CYP2E1, the cyclic voltammogram obtained under aerobic conditions displayed a reduction peak at *E*_c_ −0.228 ± 0.005 V (Figure S5 in the Supplementary Materials). Consequently, the reduction potential of the CYP2E1 heme iron ion under aerobic conditions exceeded by 73 mV the potential observed for CYP2E1 under anaerobic conditions. Similarly, for cyt *b*_5_, the cyclic voltammogram registered under aerobic conditions revealed a reduction peak at *E*_c_ −0.200 ± 0.007 V (Figure S6 in the Supplementary Materials), which was 88 mV more positive than the reduction potential of this protein under anaerobic conditions. When examining the CYP2E1 + cyt *b*_5_ mixture, the reduction peak potential, *E*_c_, was determined to be −0.214 ± 0.023 (Figure S7 in the Supplementary Materials), representing a 96 mV increase compared with the reduction peak *E*_c_ registered under anaerobic conditions. Although cyclic voltammetry failed to detect the redox process for CPR under anaerobic conditions, the cyclic voltammogram under aerobic conditions indicated a reduction peak at *E*_c_ −0.429 ± 0.024 V and an oxidation peak at *E*_a_ −0.396 ± 0.006 V (Figure S8 in the Supplementary Materials). These peaks represent the reduction and oxidation processes of the flavin prosthetic groups of CPR. Notably, the potential values obtained in this case closely align with the corresponding potential values registered using differential pulse voltammetry for CPR under anaerobic conditions. Thus, from the obtained characteristics, it can be inferred that the reduction peak at *E*_c_ −0.262 ± 0.007 V, observed for Bactosomes under aerobic conditions, likely corresponds to the reduction of the heme iron ions in both CYP2E1 and cyt *b*_5_, while the reduction peak at *E*_c_ −0.418 ± 0.017 V corresponds to the reduction of the flavin prosthetic groups of CPR. Furthermore, the presence of two reduction peaks for Bactosomes under aerobic conditions and a single reduction peak under anaerobic conditions can be explained. The shift of the reduction potentials for recombinant CYP2E1 and cyt *b*_5_ to a more positive range in the presence of oxygen also accounts for the observation that, under aerobic conditions, the reduction peaks for the heme iron ions in these two hemoproteins and the flavin prosthetic groups of CPR become distinguishable when compared with anaerobic conditions. It is worth noting that, under aerobic conditions, there is no oxidation peak for CPR in Bactosomes, unlike recombinant CPR, which exhibits an oxidation peak. This discrepancy is likely attributable to the fact that, in Bactosomes, following the electrochemical reduction of flavin prosthetic groups, electrons from CPR are transferred to CYP2E1 and/or cyt *b*_5_, both of which have more positive potential values than CPR. In other words, this mechanism involves intermolecular electron transfer, similar to natural cytochrome P450-containing systems (electron donor → CPR → cytochrome P450/cyt *b*_5_).

### 3.2. Electrochemical Analysis of Chlorzoxazone and 6-Hydroxychlorzoxazone

**Figure 4:**
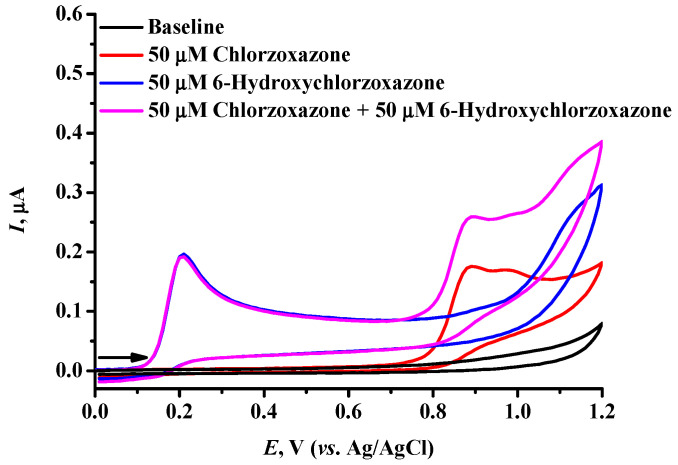
Cyclic voltammograms of SPEs registered in a 100 mM potassium phosphate buffer, pH 7.4, containing 50 mM NaCl (▬) and 50 μM chlorzoxazone (▬) or 50 μM 6-hydroxychlorzoxazone (▬), or a mixture of 50 μM chlorzoxazone and 50 μM 6-hydroxychlorzoxazone (▬). The voltammograms were registered at a 100 mV/s scan rate.

To develop a procedure for electrochemical quantification of 6-hydroxychlorzoxazone formed during CYP2E1-mediated chlorzoxazone hydroxylation, we conducted a study of the electrochemical properties of chlorzoxazone and 6-hydroxychlorzoxazone, as well as their equimolar mixture, using cyclic voltammetry in 100 mM potassium phosphate buffer at pH 7.4, containing 50 mM NaCl (Figure 4). At a concentration of 50 μM, chlorzoxazone exhibits an oxidation peak at 0.892 V and a peak at 0.972 V.

**Figure 5:**
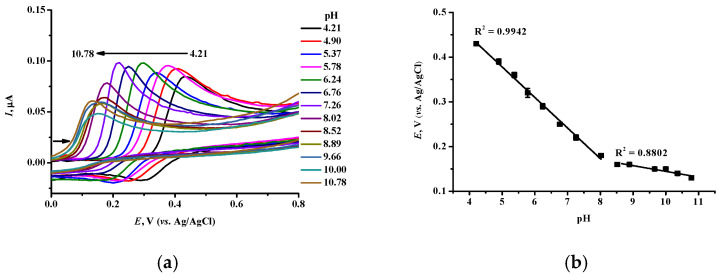
Cyclic voltammograms of SPEs registered in 100 mM potassium phosphate supporting solution, containing 50 mM NaCl, 1% ethanol (*v*/*v*), and 50 μM 6-hydroxychlorzoxazone, in the pH range of 4.21–10.78 (**a**). The dependence of 6-hydroxychlorzoxazone oxidation peak potential on pH (**b**). The values are the means from three experiments ± standard deviations. The voltammograms were registered at a 100 mV/s scan rate.

Previously, Abbar et al. investigated the electrochemical characteristics of chlorzoxazone by employing a glassy carbon electrode [55]. The authors registered the oxidation peak of chlorzoxazone at 0.9072 V (vs. Ag/AgCl) in a 200 mM phosphate buffer at pH 7.0. They also presented evidence that electrochemical oxidation of chlorzoxazone occurs with the participation of a number of electrons equivalent to the number of protons. Based on experimental data, the authors proposed a mechanism of electrochemical oxidation for chlorzoxazone, accompanied by the abstraction of one proton and one electron, followed by hydration, decarboxylation, and the formation of the final product 2-amino-4-chlorophenol. Subsequent research by Baniahmad et al. supported this proposed mechanism for the electrochemical oxidation of this compound [56]. For 6-hydroxychlorzoxazone at a concentration of 50 μM, we observed an oxidation peak at approximately 0.207 V, a smaller-amplitude peak near 0.891 V, and another peak at 1.114 V. We postulate that the 0.207 V oxidation peak may correspond to the oxidation process of the hydroxyphenyl group of 6-hydroxychlorzoxazone, while the 0.891 V and 1.114 V peaks appear to indicate oxidation following a mechanism similar to the chlorzoxazone oxidation process. To propose a possible oxidation mechanism for 6-hydroxychlorzoxazone at 0.2 V we registered cyclic voltammograms for 50 μM 6-hydroxychlorzoxazone in 60 μL of 100 mM potassium phosphate supporting solution, containing 50 mM NaCl and 1% ethanol (*v*/*v*), in the pH range of 4.21–10.78 (Figure 5).

As demonstrated in Figure 5, at increasing pH, the oxidation peak of 6-hydroxychlorzoxazone shifts to a lower potential. At the same time, a reduction peak at 0.1–0.3 V with a current approximately 5.5 times lower compared with the current of the oxidation peak can be registered. At a pH of 8.02, the reduction peak virtually disappears. The dependence of the oxidation potential on pH in the range 4.21–8.02 for 6-hydroxychlorzoxazone was linear (R^2^ = 0.9942) and followed the equation *E*, V = −(0.06833 ± 0.00197), V/pH × pH + (0.71957 ± 0.01217), V. Thus, the obtained slope was equal to 0.068 V/pH and was close to the theoretical Nernstian predicted value of 0.059 V/pH, which indicates that the number of electrons involved in the electrochemical process is equal to the number of protons. In the 8.52–10.78 pH range, the oxidation potential of 6-hydroxychlorzoxazone changed insignificantly, and, after linear approximation (R^2^ = 0.8802) of the dependence, the equation was *E*, V = −(0.01285 ± 0.00209), V/pH × pH + (0.27299 ± 0.02036), V. The break in the dependence of the oxidation potential of 6-hydroxychlorzoxazone on pH indicates it reaching a pH (8.02–8.89) close to the p*K*_a_ of the hydroxyphenyl group of the 6-hydroxychlorzoxazone molecule. This is in accordance with the p*K*_a_ value being equal to 8.55 for 2-chlorphenol [57], a fragment of the 6-hydroxychlorzoxazone structure. Additionally, pH 8.3 corresponds to chlorzoxazone’s p*K*_a_ [58] and is apparently close to that of the imino group of 6-hydroxychlorzoxazone. Thus, at pH 8.02–8.89, the number of protons involved in 6-hydroxychlorzoxazone oxidation decreases, as indicated by the insignificant changes in the oxidation potential of 6-hydroxychlorzoxazone. The number of electrons (*n*) involved in 6-hydroxychlorzoxazone oxidation can be calculated using Equation (3) [59]:

(3) $$E_{p}-E_{\frac{p}{2}}=\frac{1.857RT}{\alpha nF},$$

where *E*_p_ is the peak potential, V, $E_{\frac{p}{2}}$ is the half-peak potential, V, *R* is the gas constant, *T* is the temperature, K, and *α* is the electron transfer coefficient.

**Scheme 2:**
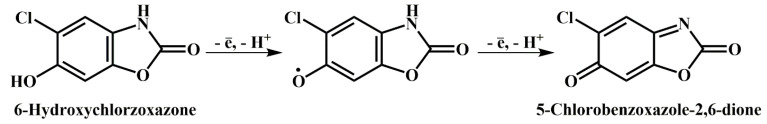
The proposed mechanism of electrochemical oxidation for 6-hydroxychlorzoxazone.

Assuming *α* equal to 0.5, the mean *n* in the pH range of 4.21–10.78 was calculated as 1.9, while the mean number of protons involved in 6-hydroxychlorzoxazone oxidation in the pH range of 4.21–8.02 was calculated, accounting for the slope of the dependence of the potential peak on pH, as 2.3. Thus, we propose that the oxidation mechanism for 6-hydroxychlorzoxazone is associated with the removal of two electrons and two protons and formation of 5-chlorobenzoxazole-2,6-dione as the final product (Scheme 2).

In an equimolar mixture of chlorzoxazone and 6-hydroxychlorzoxazone, oxidation peaks were identified at 0.207 V, 0.896 V, 0.980 V, and 1.120 V. The 0.207 V peak displayed potential and current characteristics resembling those of the 6-hydroxychlorzoxazone oxidation peak in the absence of chlorzoxazone. The 0.896 V, 0.980 V, and 1.120 V peaks represent the oxidation of 6-hydroxychlorzoxazone and the oxidation of chlorzoxazone. Thus, we have demonstrated the feasibility of quantifying 6-hydroxychlorzoxazone by monitoring its electrochemical oxidation peak in the presence of chlorzoxazone.

**Figure 6:**
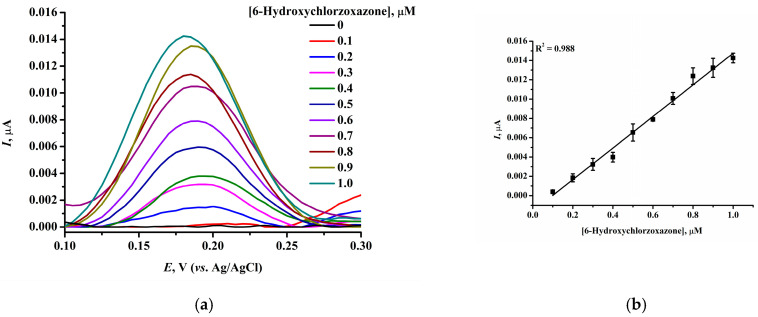
Square-wave voltammograms of SPEs registered in 100 mM potassium phosphate buffer, pH 7.4, containing 50 mM NaCl and different concentrations (0–1 μM) of 6-hydroxychlorzoxazone (**a**); and dependence of the oxidation peak current on concentrations of 6-hydroxychlorzoxazone (**b**). Mean values ± standard deviations from 3–5 independent experiments are presented. Frequency 25 Hz, amplitude 40 mV, step potential 5 mV.

We used square-wave voltammetry (SWV) to quantify 6-hydroxychlorzoxazone. The peak oxidation of the hydroxyphenyl group of 6-hydroxychlorzoxazone was detected in the potential range around 0.2 V (Figure 6a). The dependence of the oxidation peak current on the concentration of 6-hydroxychlorzoxazone (in the range 0.1–1 μM) in 100 mM potassium phosphate buffer, pH 7.4, containing 50 mM NaCl, was linear (R^2^ = 0.988) and was described by the equation: *I*, μA = (0.01635 ± 0.00059), µA/µM × [6-Hydroxychlorzoxazone], µM—(0.00161 ± 0.00036), µA (Figure 6b). The sensitivity was determined to be 0.016 µA/µM. The limit of detection, calculated as the threefold standard deviation of the average current value in the region of a 6-hydroxychlorzoxazone oxidation potential of about 0.2 V, was determined as 0.11 μM. The relative standard deviation (RSD%) of the oxidation currents for 6-hydroxychlorzoxazone was calculated as 14%. Thus, we have developed a procedure for the quantitation of 6-hydroxychlorzoxazone based on its direct electrochemical oxidation on unmodified SPEs. Subsequently, we utilized this procedure to detect and quantify this metabolite, allowing for the evaluation of the catalytic activity of Bactosomes towards chlorzoxazone.

### 3.3. Activity Evaluation and Determination of the Steady-State Kinetic Parameters of Bactosomes Immobilized on SPEs

**Figure 7:**
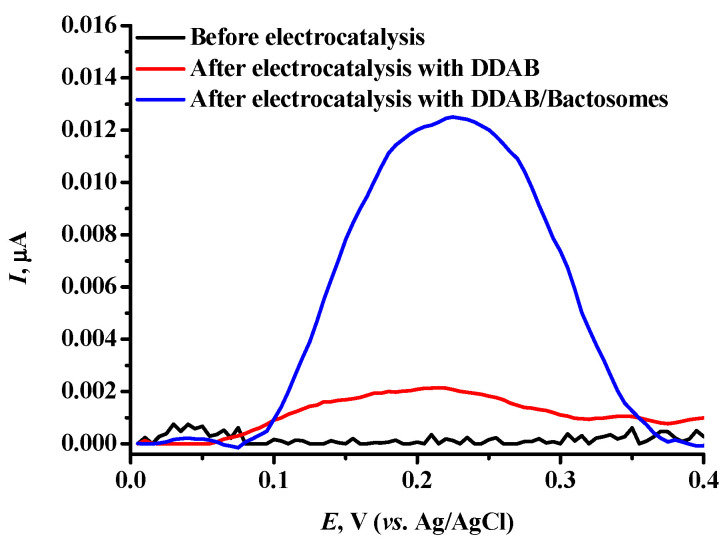
Square-wave voltammograms of SPEs registered before (▬) or after the electrocatalytic reaction in 100 mM potassium phosphate buffer, pH 7.4, containing 50 mM NaCl and 500 μM chlorzoxazone, for 60 min at a fixed potential of −0.55 V of a working electrode, modified with DDAB (▬) and with immobilized Bactosomes (▬). Frequency 25 Hz, amplitude 40 mV, step potential 5 mV.

We assessed the potential for evaluating the catalytic activity of CYP2E1 in Bactosomes using the developed bielectrode system. To assess the catalytic activity of Bactosomes immobilized on DDAB-modified SPEs towards chlorzoxazone, we employed square-wave voltammetry to detect the resulting 6-hydroxychlorzoxazone. The electrocatalytic reaction was conducted under a fixed working electrode potential of −0.55 V, at which the electrochemical reduction of the components of Bactosomes took place. This reaction was carried out for 60 min in 100 mM potassium phosphate buffer at pH 7.4, containing 50 mM NaCl and 500 μM chlorzoxazone. As a control experiment, we conducted an electrocatalytic reaction in the presence of 500 μM chlorzoxazone under similar conditions, in this case using a DDAB-modified electrode without immobilized Bactosomes. As depicted in Figure 7, following the electrocatalytic reaction with Bactosomes immobilized on the electrode, we observed an oxidation peak within the potential range corresponding to 6-hydroxychlorzoxazone electrochemical oxidation. Furthermore, after conducting the electrocatalytic reaction with an electrode without Bactosomes, an oxidation peak was also observed in the same potential range, corresponding to 6-hydroxychlorzoxazone electrochemical oxidation, albeit with a current value approximately six times lower compared with the current value registered after the electrocatalytic reaction involving chlorzoxazone with Bactosomes immobilized on the electrode. This supports the predominant role of immobilized Bactosomes in 6-hydroxychlorzoxazone formation. As shown on Figure S9 in the Supplementary Materials, at a 500 μM concentration of chlorzoxazone, the amount of 6-hydroxychlorzoxazone produced during electrocatalysis with the Bactosomes immobilized on electrodes depended linearly on the reaction time in the range of 30–90 min (R^2^ = 0.9938). The activity of CYP2E1-containing Bactosomes immobilized on an electrode towards 500 μM chlorzoxazone was calculated to be 1.47 ± 0.05 min^−1^.

We compared the activity value for Bactosomes on the electrode obtained using the electrochemical quantitation of 6-hydroxychlorzoxazone with that obtained using 6-hydroxychlorzoxazone quantitation by thin-layer chromatography followed by spectral analysis (Figure S10 in the Supplementary Materials). Figure S10 in the Supplementary Materials demonstrates that at approximately 285 nm, absorbance peaks attributed to 6-hydroxychlorzoxazone can be registered both after the elution of a spot containing 2.5 nmol of 6-hydroxychlorzoxazone and after the elution of a spot obtained by thin-layer chromatographic separation of the incubation mixture after the electrocatalytic reaction in 100 mM potassium phosphate buffer, pH 7.4, containing 50 mM NaCl and 500 μM chlorzoxazone for 90 min at a fixed potential of −0.55 V of a working electrode, modified with DDAB and with immobilized Bactosomes. From the calibration dependence of the absorbance maximum of 6-hydroxychlorzoxazone at the wavelength range of 280–300 nm on the compound’s concentration in the standard solutions, the amount of 6-hydroxychlorzoxazone produced during the electrocatalytic reaction was determined, which corresponded to the activity of Bactosomes immobilized on an electrode, equal to 1.50 ± 0.61 min^−1^. Thus, the values of the activities of the Bactosomes immobilized on an electrode obtained using two different methods of 6-hydroxychlorzoxazone quantitation corresponded well.

**Figure 8:**
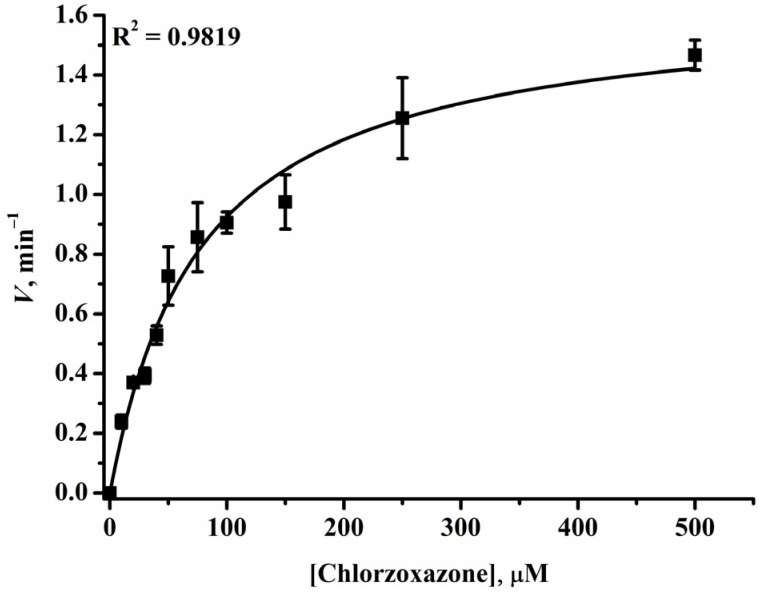
The dependence of the initial rate of 6-hydroxychlorzoxazone formation by Bactosomes immobilized on SPEs on chlorzoxazone concentration. Mean values ± standard deviations from at least five independent experiments are presented.

In order to determine the steady-state kinetic parameters of CYP2E1 in Bactosomes immobilized on SPEs towards chlorzoxazone, we assessed the initial rate of 6-hydroxychlorzoxazone formation as a function of chlorzoxazone concentration in the electrochemical system. 6-hydroxychlorzoxazone was quantified using square-wave voltammetry. The dependence of the initial rate of 6-hydroxychlorzoxazone formation on chlorzoxazone concentration (Figure 8) displayed a hyperbolic profile (R^2^ = 0.9819) and was described by the Michaelis–Menten equation (Equation (2)). The *V*_max_ and *K*_M_ values were 1.64 ± 0.08 min^−1^ and 78 ± 9 μM, respectively. The catalytic efficiency, expressed as *V*_max_/*K*_M_, was calculated to be 0.021 min^−1^/μM. Notably, the obtained *V*_max_ value was approximately 5.7 times lower than the parameter reported by the manufacturer for the steady-state kinetics of CYP2E1 in Bactosomes towards chlorzoxazone (9.3 min^−1^), while the *K*_M_ value was virtually the same (75 μM), so the catalytic efficiency in the electrochemical system was approximately six times lower than in the manufacturer’s report sheet, which could indicate conformational changes caused by immobilization. In addition, Peter et al. previously investigated the 6-hydroxylase activity of human liver microsomes from different donors towards chlorzoxazone and obtained an average *K*_M_ value equal to 39 ± 7 μM [42]. Eagling et al. and Lucas et al. reported the *K*_M_ values 59.2 ± 5.7 μM [60] and 53–74 μM [61] for 6-hydroxylation of chlorzoxazone by human liver microsomes, respectively. Ono et al. have also investigated the chlorzoxazone 6-hydroxylase activity of human liver microsomes, but with a recombinant vaccinia virus containing CYP2E1 cDNA, and obtained the *K*_M_ value of 232 µM [62].

We estimated the analytical characteristics of the developed system for CYP2E1 activity determination. The RSD% value for the activities across five independent experiments was 8%, indicating satisfactory reproducibility for the system. The RSD% value for the activities determined within one sample using the same electrode (repeatability) was found to be 21.3%. The relatively high value for this parameter could be explained by the fact that disposable electrodes were used for 6-hydroxychlorzoxazone quantification. We also studied the stability of the electrodes with Bactosomes. We found that the activity of immobilized Bactosomes continues to be stable on the fifth day after immobilization (Figure S11 in the Supplementary Materials).

### 3.4. Study of the Influence of the Applied Reduction Potential on the Electrocatalytic Activity of Bactosomes

In this part of the study, our objective was to explore the influence of CPR on the electrocatalytic activity of Bactosomes towards chlorzoxazone. We hypothesized that when electrons are transferred from the electrode to CPR and then from CPR to CYP2E1/cyt *b*_5_, the initial rate of chlorzoxazone hydroxylation will be higher than the initial rate of chlorzoxazone hydroxylation when reducing CYP2E1/cyt *b*_5_ alone. To test this hypothesis, we conducted electrocatalytic reactions with Bactosomes immobilized on an electrode in 100 mM potassium phosphate buffer, pH 7.4, containing 50 mM NaCl, 280 units/mL catalase, and 150 μM chlorzoxazone, at reduction potentials of −0.55 V and −0.35 V.

**Figure 9:**
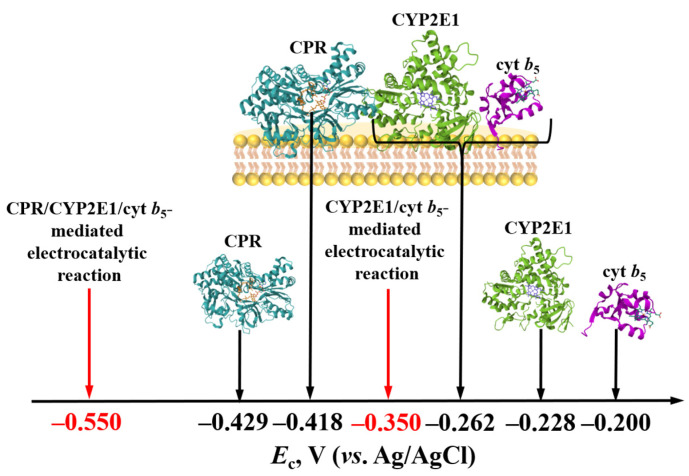
The reduction potentials of the components of Bactosomes, recombinant CYP2E1, cyt *b*_5_, and CPR, and the potentials applied to the working electrodes with immobilized Bactosomes during the electrocatalytic reactions towards chlorzoxazone (for the CPR/CYP2E1/cyt *b*_5_-mediated electrocatalytic reaction and for the CYP2E1/cyt *b*_5_-mediated electrocatalytic reaction).

The reduction potential of −0.55 V displayed a more negative value in comparison with the reduction peak potentials observed in the cyclic voltammogram registered for Bactosomes under aerobic conditions (as shown in Figure 3). Therefore, we postulated that when conducting an electrocatalytic reaction at this fixed potential, the electron flow from the electrode would be transferred to both CPR and CYP2E1/cyt *b*_5_. At the potential of −0.35 V, which is more positive than the CPR reduction peak but more negative than the CYP2E1/cyt *b*_5_ reduction peak, electrons from the electrode should predominantly be transferred towards CYP2E1/cyt *b*_5_. Figure 9 provides a visual representation of the reduction potentials of the components of Bactosomes, recombinant CYP2E1, cyt *b*_5_, and CPR, along with the potentials applied to the working electrodes during the electrocatalytic reactions (−0.55 V for the CPR/CYP2E1/cyt *b*_5_-mediated electrocatalytic reaction and −0.35 V for the CYP2E1/cyt *b*_5_-mediated electrocatalytic reaction).

**Figure 10:**
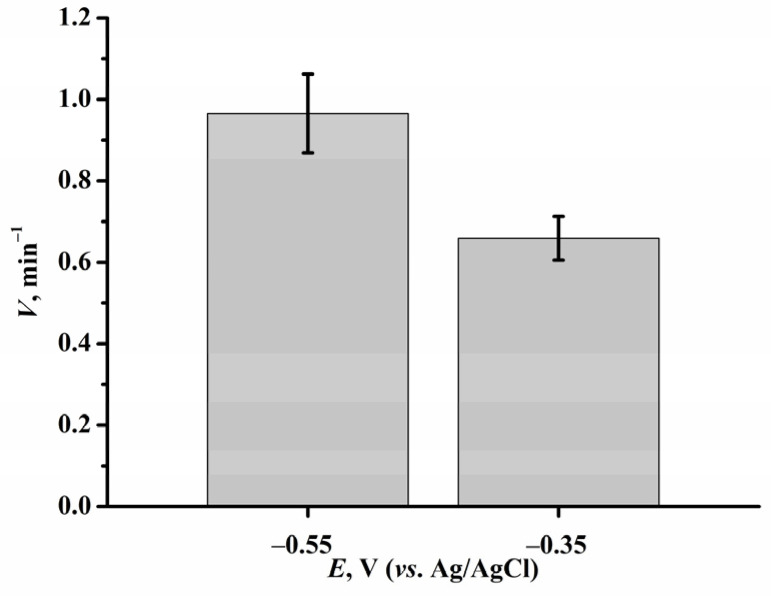
Dependence of the initial reaction rate of 6-hydroxychlorzoxazone formation on the applied reduction potential for Bactosomes immobilized on the electrode. The electrocatalytic reaction was carried out in 100 mM potassium phosphate buffer, pH 7.4, containing 50 mM NaCl, 280 units/mL catalase, and 150 μM chlorzoxazone. Mean values ± standard deviations from 3–5 independent experiments are presented.

As expected, the initial rate of formation of 6-hydroxychlorzoxazone is approximately 1.5 times greater when the electrocatalytic reaction is carried out at −0.55 V compared with the initial rate of 6-hydroxychlorzoxazone formation when the electrocatalytic reaction is carried out at −0.35 V (Figure 10). These results may indicate an activating role of CPR towards CYP2E1 because the latter simultaneously receives electrons from both the electrode and CPR.

It is worth noting that the electrocatalytic activity of Bactosomes towards chlorzoxazone in the presence of catalase at a reduction potential of −0.55 V was 0.97 min^−1^ and did not differ from the electrocatalytic activity of Bactosomes in the absence of catalase (0.97 min^−1^), suggesting that hydrogen peroxide does not contribute significantly to the formation of 6-hydroxychlorzoxazone. However, Nerimetla et al. registered a significant increase in the activity of CYP2C9 or CYP3A4-containing Bactosomes in the presence of catalase, which, according to the authors, may be due to the prevention of damage by reactive oxygen species to membrane-bound CYP and/or CPR [27]. In our case, the electrochemical formation of reactive oxygen species does not appear to significantly contribute to damage to membrane-bound CYP and CPR in Bactosomes.

## 4. Conclusions

We developed a bielectrode system for assessing the activity of CYP2E1 towards its marker substrate, chlorzoxazone. Our approach involved the utilization of SPEs modified with DDAB to immobilize Bactosomes. We assumed that this immobilization method involved interaction between Bactosomes and the modified surface of the working electrode, apparently driven by both hydrophobic and electrostatic forces. Using cyclic voltammetry under anaerobic conditions, we observed reduction and oxidation peaks for Bactosomes, seemingly corresponding to redox processes of the heme iron ions of both CYP2E1 and cyt *b*_5_. Moreover, using a more sensitive differential pulse voltammetry method compared with cyclic voltammetry, we managed to capture reduction and oxidation peaks associated with the flavin prosthetic groups of CPR. Under aerobic conditions, cyclic voltammetry registered a reduction peak, likely corresponding to the reduction of heme iron ions of CYP2E1 and cyt *b*_5_, along with a reduction peak corresponding to the flavin prosthetic groups of CPR. The absence of the CPR oxidation peak in aerobic conditions may imply intermolecular electron transfer from the electrochemically reduced form of CPR to the heme iron ions of CYP2E1/cyt *b*_5_. Hence, our data suggest that electron transfer from the electrode takes place both to CYP2E1 and cyt *b*_5_, as well as to CPR. Employing the developed method for quantification of 6-hydroxychlorzoxazone, based on recordings of the oxidation current of this metabolite by means of square-wave voltammetry, we calculated the steady-state kinetic parameters for the chlorzoxazone hydroxylation reaction with Bactosomes immobilized on an electrode. The obtained *K*_M_ value closely matched the parameter reported by the manufacturer of the Bactosomes; however, the *V*_max_ value in the electrochemical system was approximately six times lower than in the manufacturer’s report sheet, which could indicate conformational changes caused by immobilization. In conclusion, we have demonstrated that when an electrocatalytic reaction is performed at a fixed working electrode potential, at which both CYP2E1/cyt *b*_5_ and CPR are simultaneously reduced, the activity of Bactosomes towards chlorzoxazone is approximately 1.5 times as high as that achieved at the potential primarily associated with the electrochemical reduction of CYP2E1/cyt *b*_5_.

The developed bielectrode system could be used in pharmacological investigations related to the assessment of CYP2E1 activity. A notable advantage of this bielectrode approach for assessing the activity of Bactosomes immobilized on SPEs is the elimination of the need for multi-step isolation of the catalytic reaction metabolites. Furthermore, employing membrane-bound cytochrome P450 enzymes for electrode immobilization promotes a more stable electrochemical system. As a limitation of the developed approach, it could be noted that, during investigations with inhibitors of CYP2E1, there is a risk of the interference of the oxidation potential of 6-hydroxychlorzoxazone with that of the inhibitor. In order to avoid this, chromatographic separation of the reaction products can be employed. The developed approach could be used with other CYP2E1 substrates, but, in that case, we would recommend study of the electrochemical properties of the substrates and possible products beforehand. Future development of similar bielectrode systems for evaluating the catalytic activity of individual cytochrome P450 isoforms holds promise in the field of bioelectrochemistry.

## Figures and Tables

**Table 1:** Electrochemical characteristics of Bactosomes, CYP2E1, cyt *b*_5_, the CYP2E1 + cyt *b*_5_ mixture, and CPR registered under anaerobic conditions.

Immobilized Component	*E*_c_, V	*E*_a_, V	*E*_m_, V	Method
Bactosomes	−0.345 ± 0.006	−0.251 ± 0.013	−0.298 ± 0.010	CV
	−0.307 ± 0.012	−0.261 ± 0.020	−0.284 ± 0.011	DPV
	−0.454 ± 0.038	−0.410 ± 0.023	−0.432 ± 0.026	
CYP2E1	−0.301 ± 0.007	−0.263 ± 0.008	−0.282 ± 0.005	CV
cyt *b*_5_	−0.288 ± 0.009	−0.222 ± 0.017	−0.255 ± 0.005	CV
CYP2E1 + cyt *b*_5_	−0.310 ± 0.014	−0.242 ± 0.007	−0.276 ± 0.011	CV
CPR	−0.421 ± 0.003	−0.402 ± 0.012	−0.412 ± 0.005	DPV

The mean values from 3–5 experiments ± standard deviations are presented. CVs were registered at a scan rate of 50 mV/s.

## Data Availability

Data are contained within the article and Supplementary Materials file.

## Supplementary Materials

The following supporting information can be downloaded at: https://www.mdpi.com/2227-9059/12/1/152/s1, Figure S1: Cyclic voltammograms of SPEs modified with DDAB and immobilized CYP2E1 recorded in 100 mM potassium phosphate buffer, pH 7.4, containing 50 mM NaCl saturated with argon (anaerobic conditions) (a) and cyclic voltammogram obtained after subtracting the cyclic voltammogram of DDAB-modified SPE from the cyclic voltammogram of DDAB-modified SPE with immobilized CYP2E1 (b). The voltammograms were registered at a 50 mV/s scan rate; Figure S2: Cyclic voltammograms of SPEs modified with DDAB and immobilized cyt *b*_5_ registered in 100 mM potassium phosphate buffer, pH 7.4, containing 50 mM NaCl saturated with argon (anaerobic conditions) (a) and cyclic voltammogram obtained after subtracting the cyclic voltammogram of DDAB-modified SPE from the cyclic voltammogram of DDAB-modified SPE with immobilized cyt *b*_5_ (b). The voltammograms were registered at a 50 mV/s scan rate; Figure S3: Cyclic voltammograms of SPEs modified with DDAB and an immobilized mixture of CYP2E1 and cyt *b*_5_ registered in 100 mM potassium phosphate buffer, pH 7.4, containing 50 mM NaCl saturated with argon (anaerobic conditions) (a) and cyclic voltammogram obtained after subtracting the cyclic voltammogram of DDAB-modified SPE from the cyclic voltammogram of DDAB-modified SPE with an immobilized mixture of CYP2E1 and cyt *b*_5_ (b). The voltammograms were registered at a 50 mV/s scan rate; Figure S4: Differential pulse voltammograms of SPEs modified with DDAB (cathodic curve and anodic curve) and with immobilized CPR (cathodic curve and anodic curve) registered in 100 mM potassium phosphate buffer, pH 7.4, containing 50 mM NaCl saturated with argon (anaerobic conditions). Modulation amplitude 20 mV, step potential 5 mV, interval time 500 ms, modulation time 50 ms; Figure S5: Cyclic voltammograms of SPEs modified with DDAB and immobilized CYP2E1 registered in 100 mM potassium phosphate buffer, pH 7.4, containing 50 mM NaCl (aerobic conditions). The voltammograms were registered at a 50 mV/s scan rate; Figure S6: Cyclic voltammograms of SPEs modified with DDAB and immobilized cyt *b*_5_ registered in 100 mM potassium phosphate buffer, pH 7.4, containing 50 mM NaCl (aerobic conditions). The voltammograms were registered at a 50 mV/s scan rate; Figure S7: Cyclic voltammograms of SPEs modified with DDAB and an immobilized mixture of CYP2E1 and cyt *b*_5_ registered in 100 mM potassium phosphate buffer, pH 7.4, containing 50 mM NaCl (aerobic conditions). The voltammograms were registered at a 50 mV/s scan rate; Figure S8: Cyclic voltammograms of SPEs modified with DDAB and immobilized CPR registered in 100 mM potassium phosphate buffer, pH 7.4, containing 50 mM NaCl (aerobic conditions). The voltammograms were registered at a 50 mV/s scan rate; Figure S9: The dependence of 6-hydroxychlorzoxazone (pmol) produced on the time of electrocatalytic reaction at −0.55 V with SPEs modified with DDAB and immobilized Bactosomes at a concentration of chlorzoxazone of 500 μM.; Figure S10: 6-hydroxychlorzoxazone absorbance spectra obtained after thin-layer chromatography and elution with ethanol (600 μL) of the spot attributed to 2.5 nmol 6-hydroxychlorzoxazone (control) and the spot attributed to 6-hydroxychlorzoxazone obtained after the thin-layer chromatographic separation of the incubation mixture after the electrocatalytic reaction in 100 mM potassium phosphate buffer, pH 7.4, containing 50 mM NaCl and 500 μM chlorzoxazone for 90 min at a fixed potential of −0.55 V of a working electrode, modified with DDAB and with immobilized Bactosomes. The absorbance spectra were registered after baselining with ethanol; Figure S11: Dependence of the activity of the immobilized Bactosomes on storage time (days) after immobilization. The activity obtained on the first day was set as 100%. The presented values are means from three independent experiments ± standard deviations.
