# Supplementary material for: Bielectrode Strategy for Determination of CYP2E1 Catalytic Activity: Electrodes with Bactosomes and Voltammetric Determination of 6-Hydroxychlorzoxazone
Source: Biomedicines. 2024 Jan 8;12(1):152. doi: 10.3390/biomedicines12010152 (PMC10812958; doi:10.3390/biomedicines12010152)
Supplement: Supplementary file 1 [file biomedicines-12-00152-s001.zip › biomedicines-2756891-supplementary.pdf]

Supplementary materials

Bielectrode strategy for determination of CYP2E1 catalytic activity: electrodes with Bactosomes and voltammetric determination of 6-hydroxychlorzoxazone

Alexey V. Kuzikov [1,2,*], Rami A. Masamrekh [1,2], Tatiana A. Filippova [1,2], Anastasiya M. Tumilovich [3], Natallia V. Strushkevich [3,†], Andrei A. Gilep [1,3], Yulia Yu. Khudoklinova [2], Victoria V. Shumyantseva [1,2]

[1] Institute of Biomedical Chemistry, 10, Pogodinskaya Street, Moscow 119121, Russia; rami.masamreh@yandex.ru (R.A.M.); lipivif@gmail.com (T.A.F.); agilep@yahoo.com (A.A.G.); viktoria.shumyantseva@ibmc.msk.ru (V.V.S.)
[2] Pirogov Russian National Research Medical University, 1, Ostrovityanova Street, Moscow 117997, Russia; hudoklinova@mail.ru
[3] Institute of Bioorganic Chemistry NASB, 5 Building 2, V.F. Kuprevich Street, Minsk, 220084, Belarus; tumilovicham@iboch.by (A.M.T.); natstrush@gmail.com (N.V.S.)
* Correspondence: alexeykuzikov@gmail.com, Tel.: +74992465820
† Current address: Skoltech, 121205 Moscow, Russia.

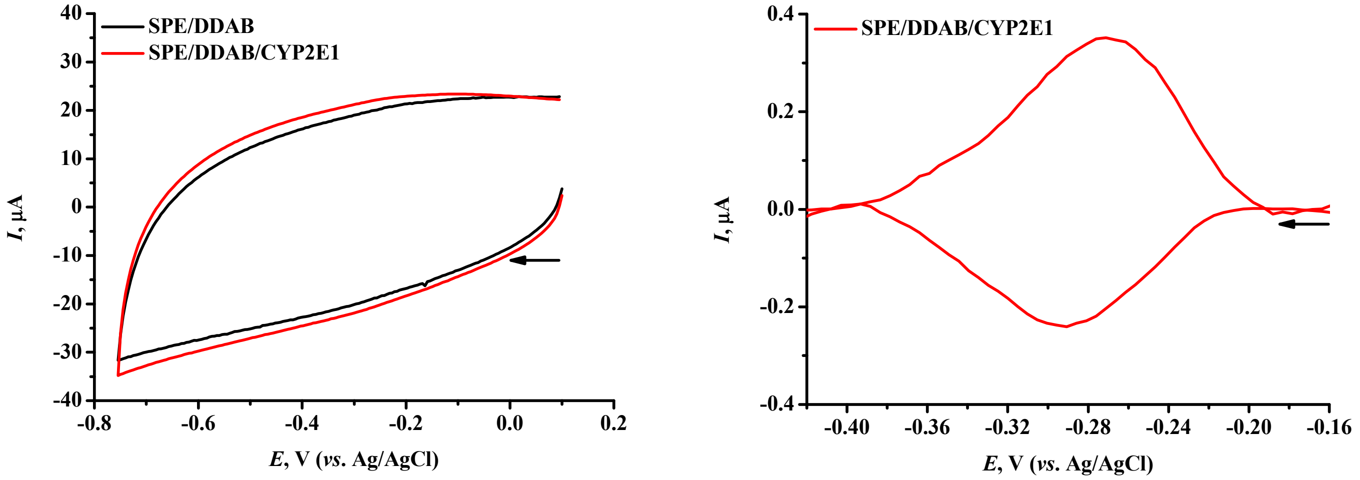

(**a**) (**b**)

Supplementary Figure S1. Cyclic voltammograms of SPEs modified with DDAB (▬) and immobilized CYP2E1 (▬) recorded in 100 mM potassium phosphate buffer, pH 7.4, containing 50 mM NaCl saturated with argon (anaerobic conditions) (**a**) and cyclic voltammogram obtained after subtracting the cyclic voltammogram of DDAB-modified SPE from the cyclic voltammogram of DDAB-modified SPE with immobilized CYP2E1 (**b**). The voltammograms were registered at a 50 mV/s scan rate.

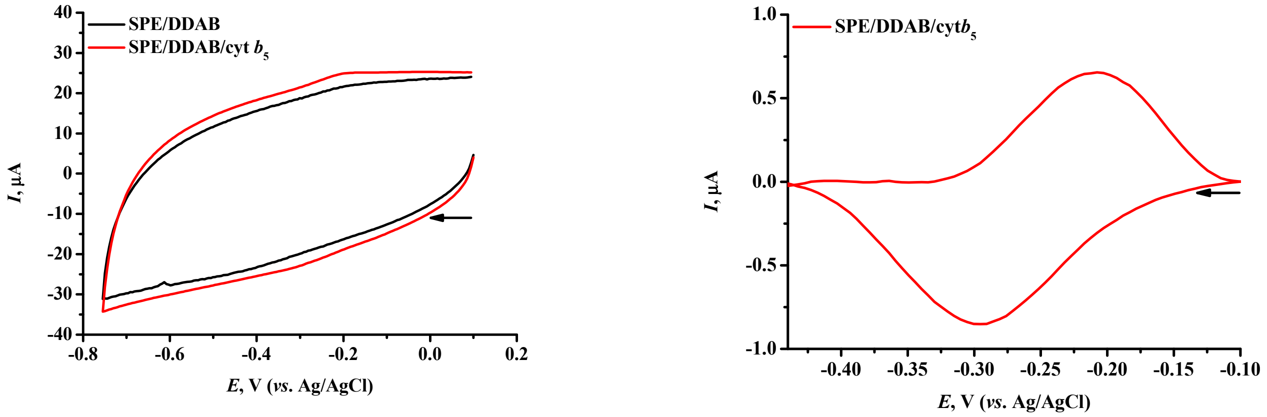

(**a**) (**b**)

Supplementary Figure S2. Cyclic voltammograms of SPEs modified with DDAB (▬) and immobilized cyt *b*5 (▬) registered in 100 mM potassium phosphate buffer, pH 7.4, containing 50 mM NaCl saturated with argon (anaerobic conditions) (**a**) and cyclic voltammogram obtained after subtracting the cyclic voltammogram of DDAB-modified SPE from the cyclic voltammogram of DDAB-modified SPE with immobilized cyt *b*5 (**b**). The voltammograms were registered at a 50 mV/s scan rate.

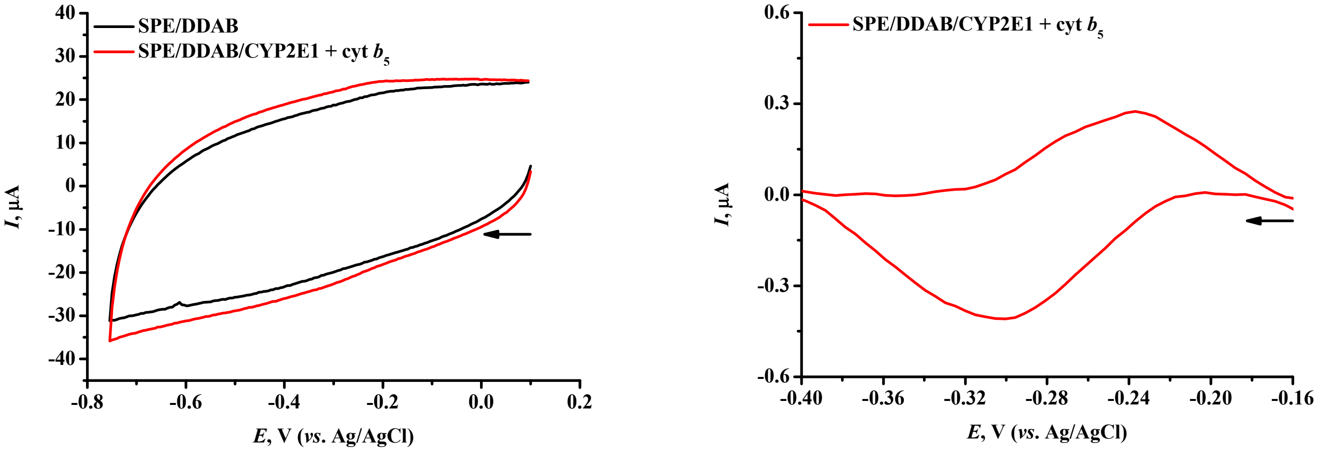

(a) (**b**)

Supplementary Figure S3. Cyclic voltammograms of SPEs modified with DDAB (▬) and immobilized mixture of CYP2E1 and cyt *b*5 (▬) registered in 100 mM potassium phosphate buffer, pH 7.4, containing 50 mM NaCl saturated with argon (anaerobic conditions) (**a**) and cyclic voltammogram obtained after subtracting the cyclic voltammogram of DDAB-modified SPE from the cyclic voltammogram of DDAB-modified SPE with an immobilized mixture of CYP2E1 and cyt *b*5 (**b**). The voltammograms were registered at a 50 mV/s scan rate.

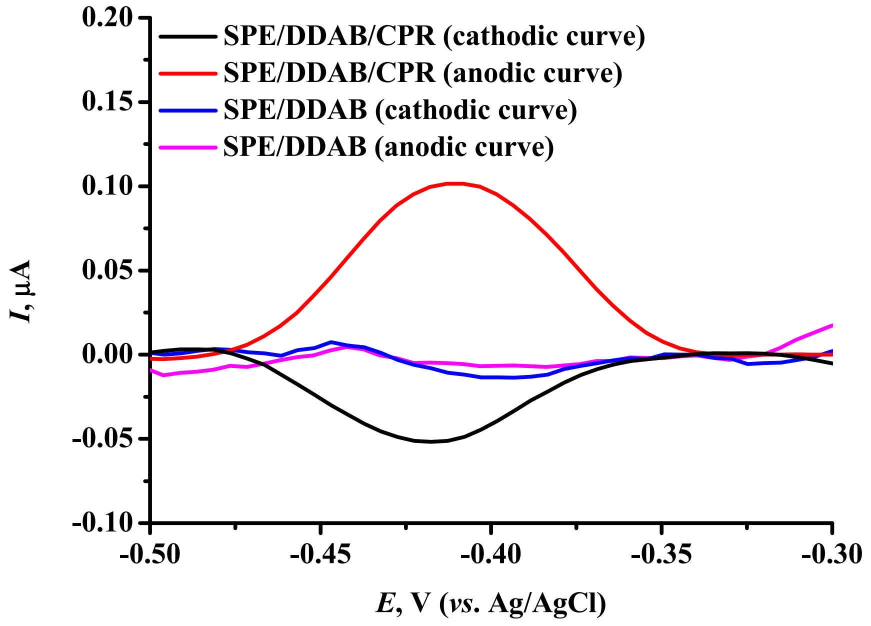

Supplementary Figure S4. Differential pulse voltammograms of SPEs modified with DDAB (cathodic curve (—) and anodic curve (—)) and with immobilized CPR (cathodic curve (—) and anodic curve (—)) registered in 100 mM potassium phosphate buffer, pH 7.4, containing 50 mM NaCl saturated with argon (anaerobic conditions). Modulation amplitude 20 mV, step potential 5 mV, interval time 500 ms, modulation time 50 ms.

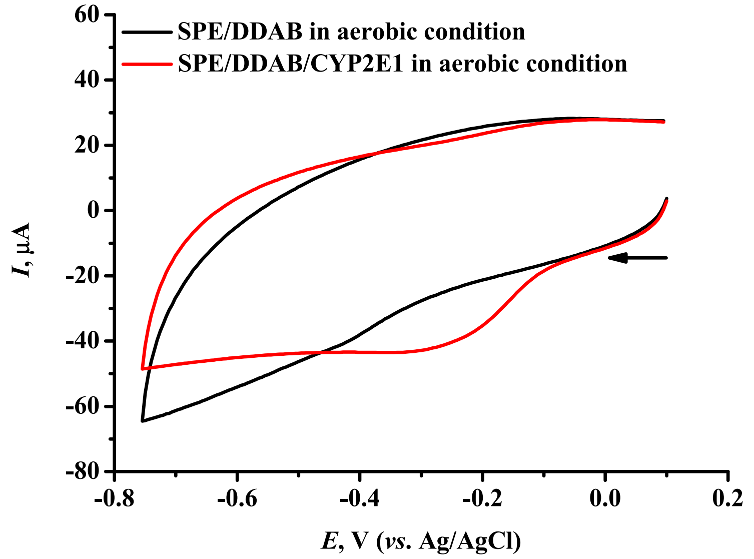

Supplementary Figure S5. Cyclic voltammograms of SPEs modified with DDAB (—) and immobilized CYP2E1 (—) registered in 100 mM potassium phosphate buffer, pH 7.4, containing 50 mM NaCl (aerobic conditions). The voltammograms were registered at a 50 mV/s scan rate.

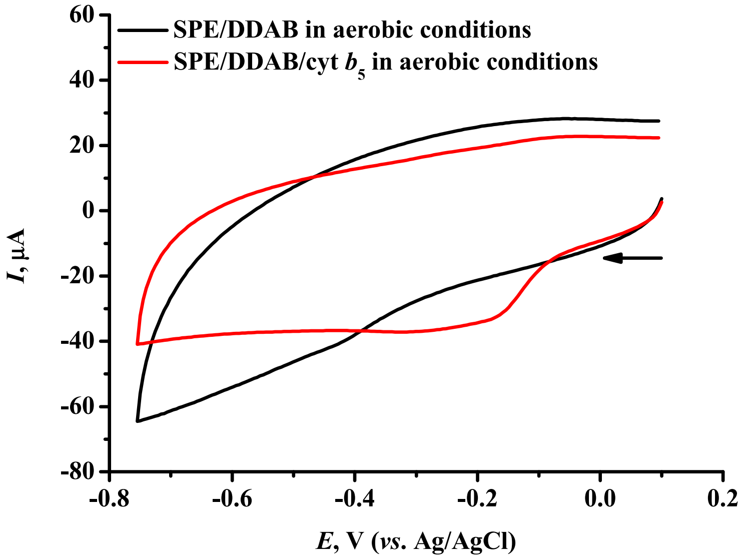

Supplementary Figure S6. Cyclic voltammograms of SPEs modified with DDAB (▬) and immobilized cyt *b*5 (▬) registered in 100 mM potassium phosphate buffer, pH 7.4, containing 50 mM NaCl (aerobic conditions). The voltammograms were registered at a 50 mV/s scan rate.

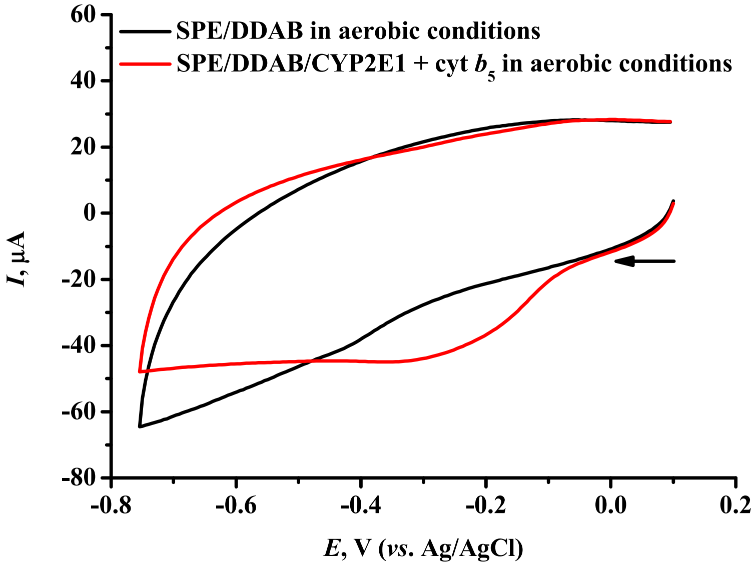

Supplementary Figure S7. Cyclic voltammograms of SPEs modified with DDAB (▬) and an immobilized mixture of CYP2E1 and cyt *b*5 (▬) registered in 100 mM potassium phosphate buffer, pH 7.4, containing 50 mM NaCl (aerobic conditions). The voltammograms were registered at a 50 mV/s scan rate.

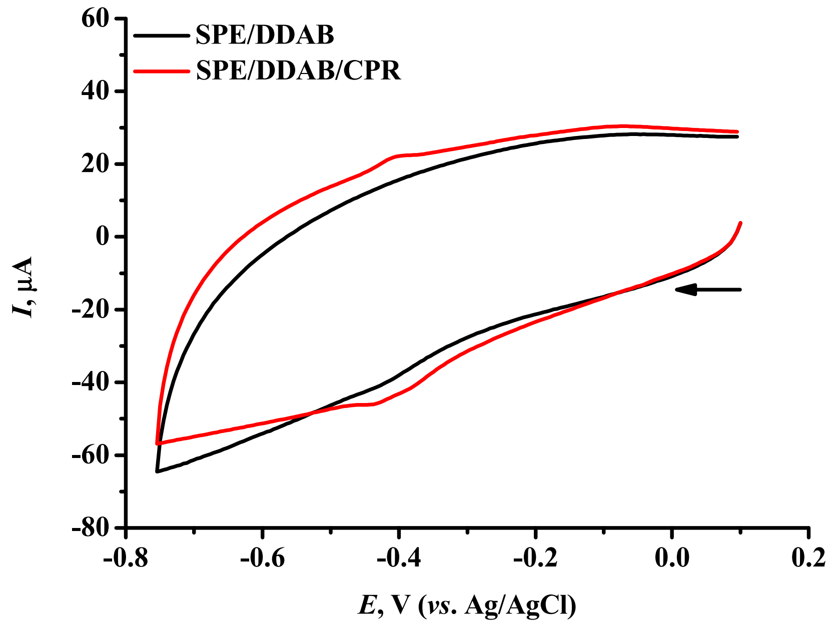

Supplementary Figure S8. Cyclic voltammograms of SPEs modified with DDAB (—) and immobilized CPR (—) registered in 100 mM potassium phosphate buffer, pH 7.4, containing 50 mM NaCl (aerobic conditions). The voltammograms were registered at a 50 mV/s scan rate.

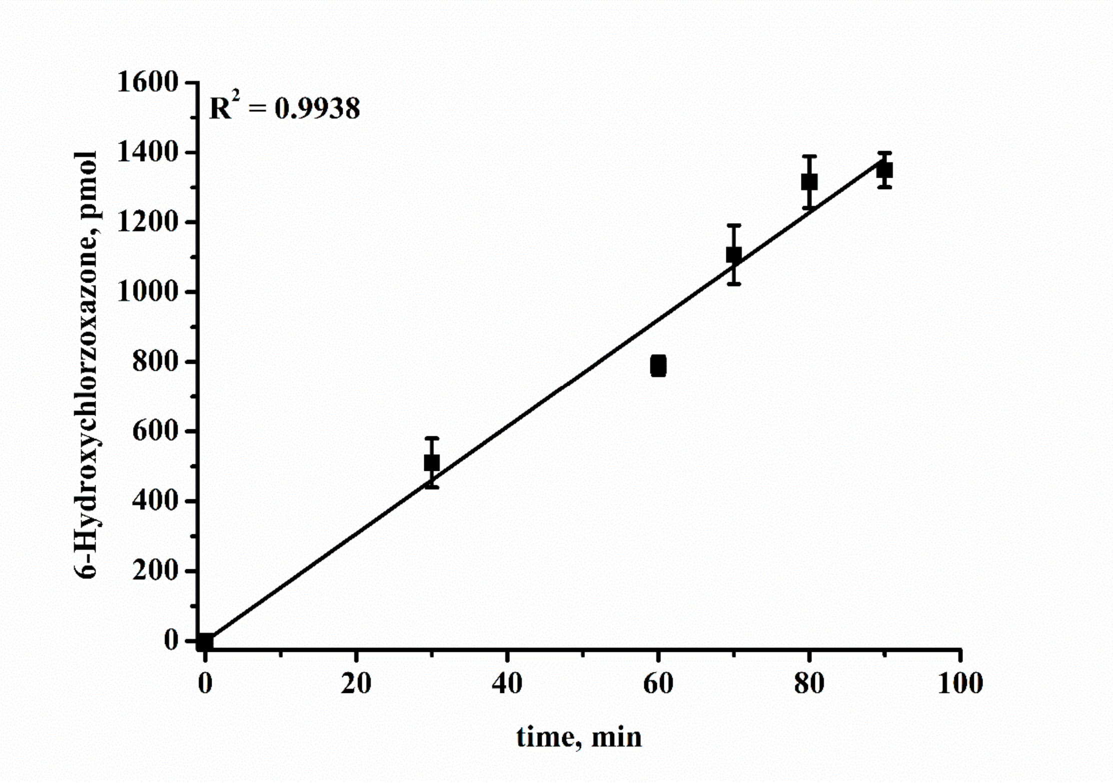

Supplementary Figure S9. The dependence of 6-hydroxychlorzoxazone (pmol) produced on the time of electrocatalytic reaction at −0.55 V with SPEs modified with DDAB and immobilized Bactosomes at a concentration of chlorzoxazone of 500 μM.

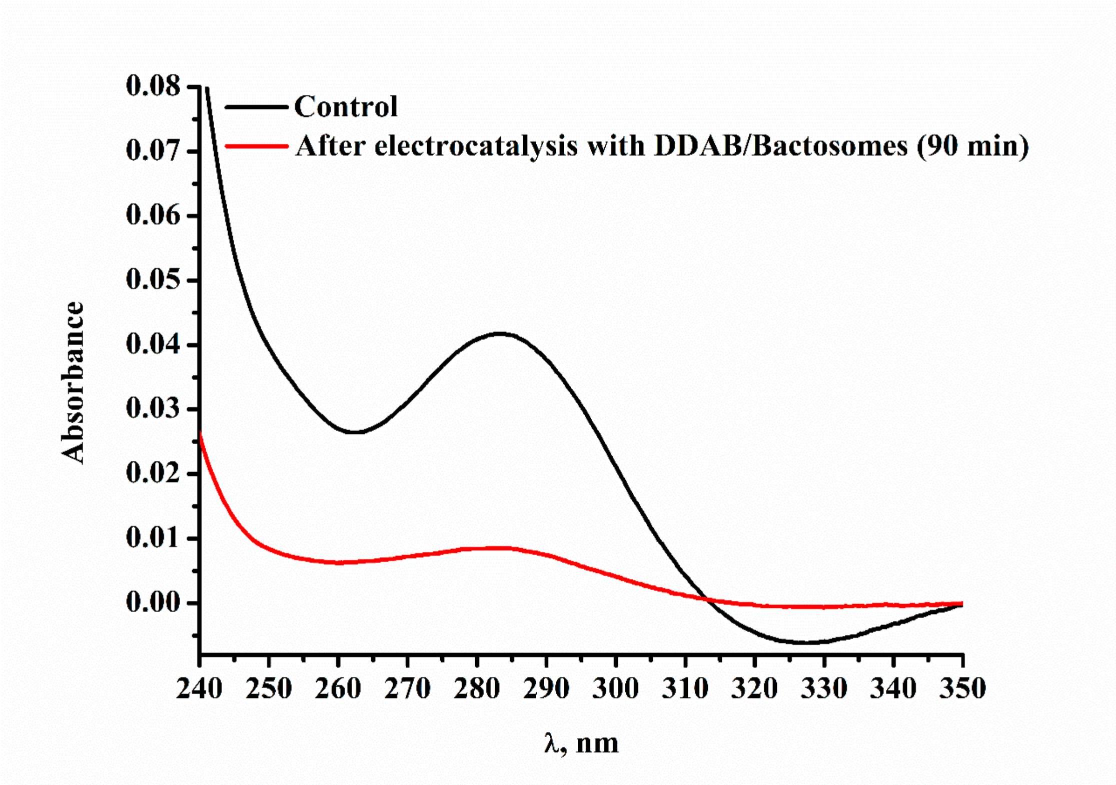

Supplementary Figure S10. 6-hydroxychlorzoxazone absorbance spectra obtained after thin-layer chromatography and elution with ethanol (600 µL) of the spot attributed to 2.5 nmol 6-hydroxychlorzoxazone (control, ▬) and the spot attributed to 6-hydroxychlorzoxazone obtained after thin-layer chromatographic separation of the incubation mixture after the electrocatalytic reaction in 100 mM potassium phosphate buffer, pH 7.4, containing 50 mM NaCl and 500 µM 6-hydroxychlorzoxazone for 90 min at a fixed potential of −0.55 V of a working electrode, modified with DDAB and with immobilized Bactosomes (▬). The absorbance spectra were registered after baselining with ethanol.

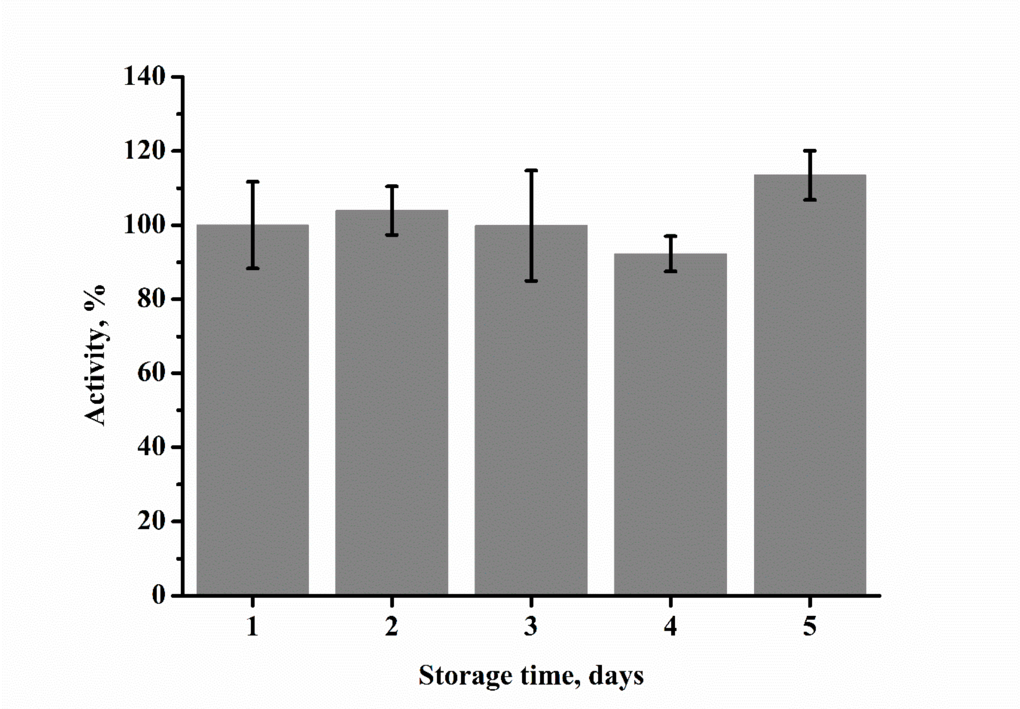

Supplementary Figure S11. Dependence of the activity of the immobilized Bactosomes on storage time (days) after immobilization. The activity obtained on the first day was set as 100%. The presented values are means from three independent experiments ± standard deviations.
